# Supplementary material for: Are indigenous territories effective natural climate solutions? A neotropical analysis using matching methods and geographic discontinuity designs
Source: PLoS One. 2021 Jul 12;16(7):e0245110. doi: 10.1371/journal.pone.0245110 (PMC8274867; doi:10.1371/journal.pone.0245110)
Supplement: S1 Appendix — (PDF) [file pone.0245110.s001.pdf]

S1 Appendix. Geospatial information and covariates

Table A. Geospatial Information and its sources by country.

**Country**	**Geospatial Information**	**Source**
All countries	Annual carbon density (2003 – 2016)	Woods Hole Research Center (Baccini et al., 2012; Baccini et al., 2017).
All countries	Elevation and slope	Shuttle Radar Topographic Mission – USGS (United States Geological Survey)
Panama	Roads, human settlements (> 5000 inhabitants)*, rivers, PAs.	STRI (“Smithsonian Tropical Research Institute”).
Panama	Titled and claimed ITs	Neotropical Ecology Laboratory (Prof. Catherin Potvin McGill University/STRI), COONAPIP (Coordinadora Nacional de los Pueblos Indígenas de Panamá).
Amazon Basin (Colombia, Ecuador, Perú, Brasil).	PAs and ITs, roads	RAISG (Red Amazónica de Información Socio-Ambiental Geo-Referenciada).
Colombia	Rivers	IGAC ("Instituto Geográfico Agustín Codazzi").
Colombia	Human settlements (> 5000 inhabitants)*	DANE (Departamento Administrativo Nacional de Estadística).
Ecuador	Human settlements (> 10 000 inhabitants)*, Rivers.	IGM ("Instituto Geográfico Militar”).

Peru	Rivers	IGN ("Instituto Geográfico Nacional").
Peru	Human settlements (> 5000 inhabitants)*	INEI ("Instituto Nacional de Estadística e Informática") and Ministry of Education.
Brazil	Human settlements (> 5000 inhabitants)*, Rivers.	IBGE ("Instituto Brasileiro de Geografía e estadística").

* The human settlements shown in the S4C Appendix includes locations with less than 5000 inhabitants.

Table B. Covariates mean differences between PAs, ITs, and OAs with other lands by country and their statistical significance from Mann Whitney U tests.

**Country**	**Land tenure**	**Roads (km)**	**Settlements (km)**	**Rivers (km)**	**Elevation (m)**	**Slope (%)**
**Panama**	**PAs**	-25.91***	-6.04***	-0.23***	213.00***	-2.00***
	**ITs**	-52.36***	-8.20***	-0.33***	-154.00***	-2.00***
	**OAs**	-109.12***	-13.86***	-0.01***	-3.00***	-3.00***
**Colombia**	**PAs**	-29.10***	-37.56***	-0.25***	12.00***	0.00***
	**ITs**	-13.92***	-62.62***	0.16*	177.00*	1.00*
	**OAs**	-28.87***	-70.74***	1.07***	186.00***	1.00***
**Ecuador**	**PAs**	-12.50***	-8.66***	-0.53***	-309.00***	-2.00***
	**ITs**	-28.49***	-16.21***	0.04***	415.00***	3.00***
	**OAs**	-23.36***	-25.11***	-0.13***	447.00***	3.00***

**Peru**	**PAs**	-28.05***	-46.95***	-0.04***	-22.00***	0.00***
	**ITs**	2.06***	5.07***	2.08***	29.00***	0.00***
	**OAs**	-29.54***	3.26***	-2.85***	235.00***	2.00***
**Brazi**	**PAs**	-27.22***	-33.14***	-3.54***	9.00***	0.00***
	**ITs**	-32.17***	-91.69***	-7.91***	-53.00***	0.00***
	**OAs**	-15.65***	-126.83***	-15.65***	-49.00***	-1.00***

*** p < 0.001, ** p < 0.01, * p < 0.05.

Table C. Coarsening Choices applied through Coarsened Exact Matching (CEM) by country across PAs, ITs, and OAs.

**Country**	**Land tenure**	**Roads (km)**	**Settlements (km)**	**Rivers (km)**	**Elevation (m)**	**Slope (%)**
Panama	PAs	0.5	0.5	0.5	50	1
	ITs	2.5	2	1	100	1.5
	OAs	1	2	1	150	1.5
Colombia	PAs	1	1	0.5	50	1.5
	ITs	2	1	1	50	1.5
	Overlapped Areas	2	2	1	100	1.5
Ecuador	PAs	1	1	1	50	2
	ITs	1	2	0.8	150	2
	OAs	0.5	2	0.7	150	2
Peru	PAs	2	1	2.5	200	2

	ITs	1	1	1	150	2
	OAs	1	1	1	150	2
Brazil	PAs	1	1	1	100	1
	ITs	1	1	1	200	1.5
	OAs	1	1	1	200	1.5
