# Supplementary material for: Are indigenous territories effective natural climate solutions? A neotropical analysis using matching methods and geographic discontinuity designs
Source: PLoS One. 2021 Jul 12;16(7):e0245110. doi: 10.1371/journal.pone.0245110 (PMC8274867; doi:10.1371/journal.pone.0245110)

S2 Appendix. Covariate balance statistics and falsification tests.

Fig A. Covariates standard mean differences between ITs, OAs, and PAs with other lands before (Pre-Match) and after matching analysis (Matched) across neotropical countries.

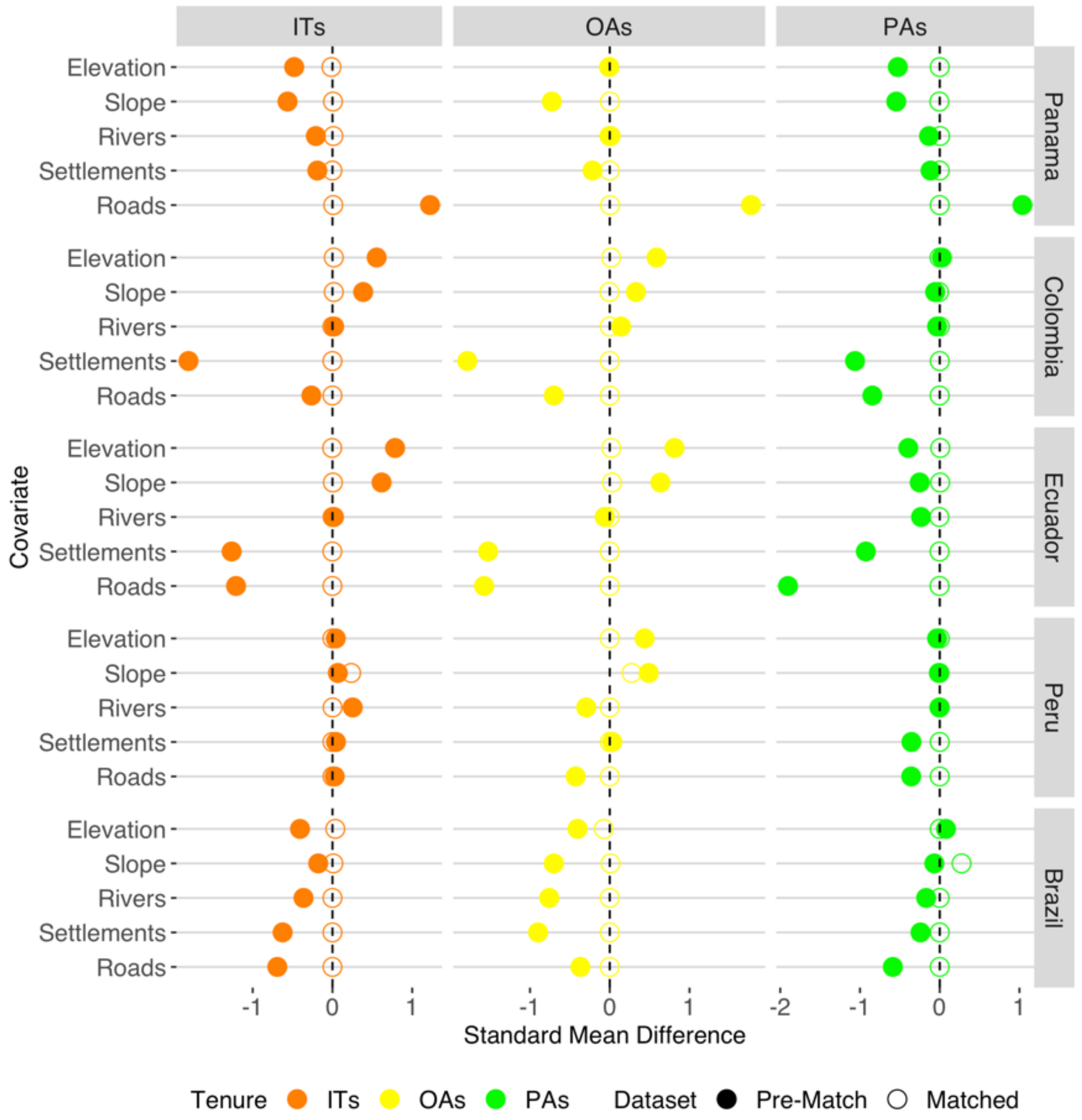

Fig B. Kolmogorov-Smirnov statistics of covariates between ITs, OAs, and PAs with other lands before (Pre-Match) and after matching analysis (Matched) in neotropical countries.

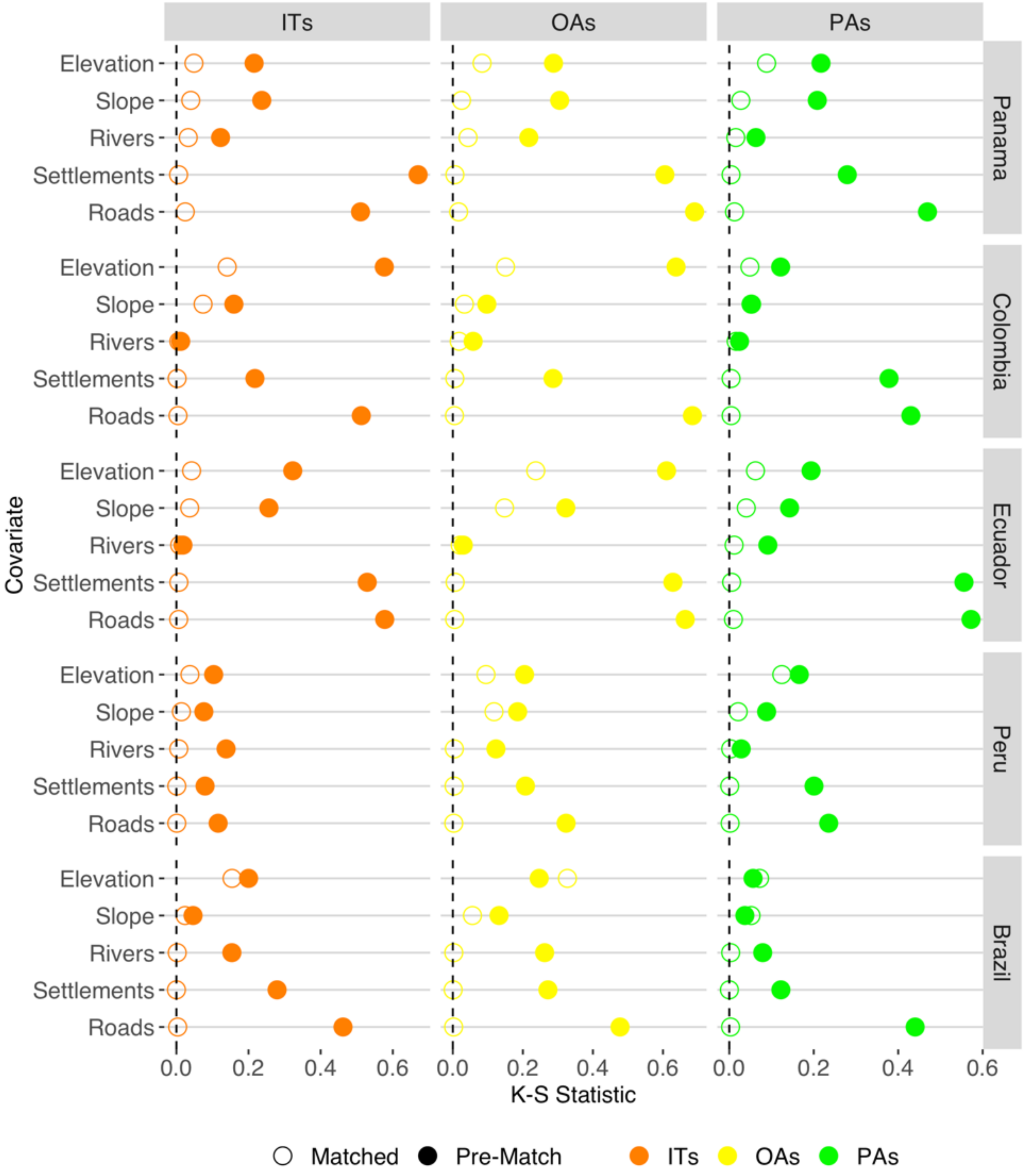

Fig C. Covariate standard mean differences before (Pre-Match) and after matching (Matched) in geographic discontinuity designs across the boundaries of ITs, OAs and PAs in neotropical countries.

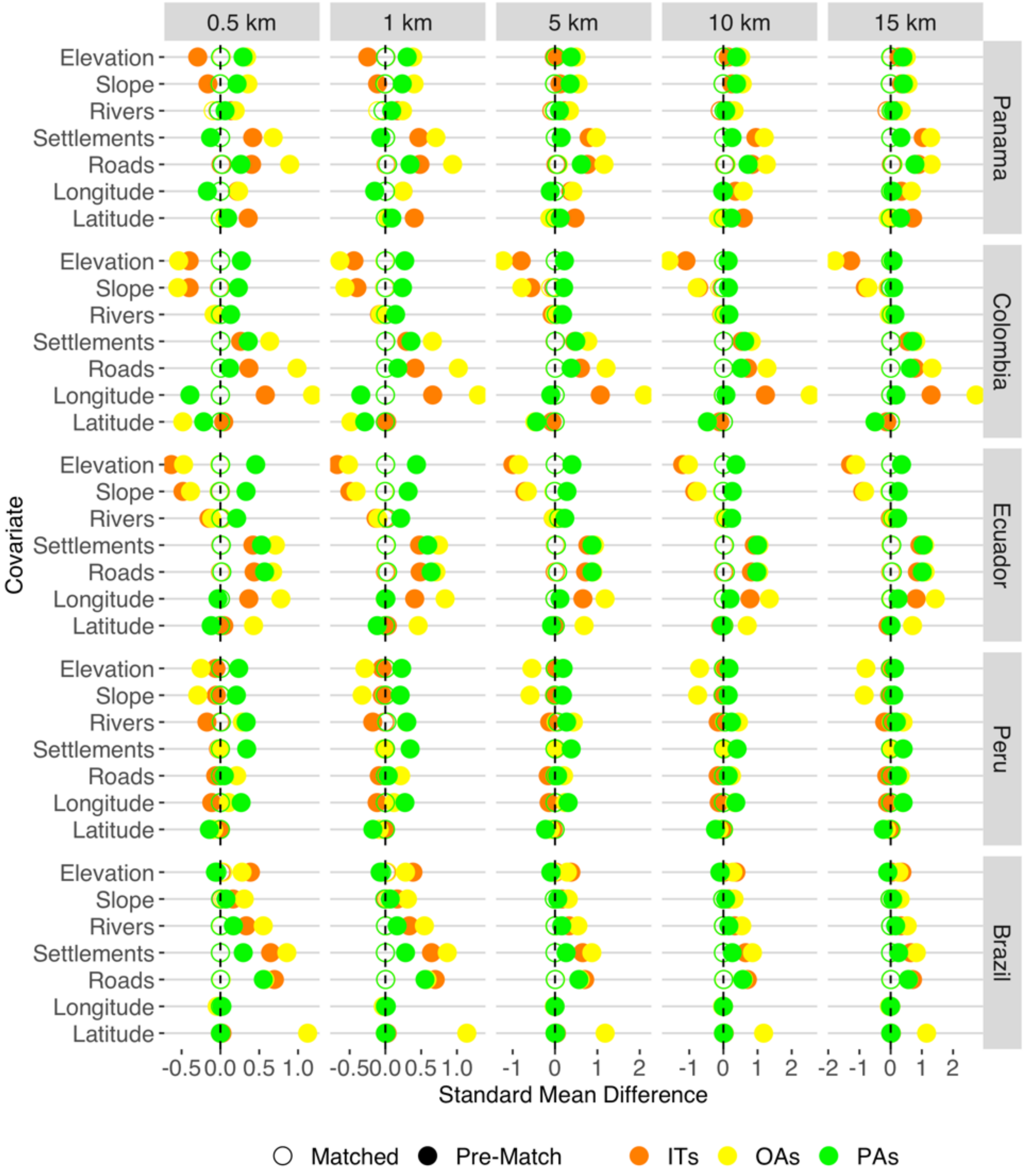

Fig D. Kolmogorov-Smirnov statistics of covariates before (Pre-Match) and after matching (Matched) in geographic discontinuity designs across the boundaries of ITs, OAs and PAs in neotropical countries.

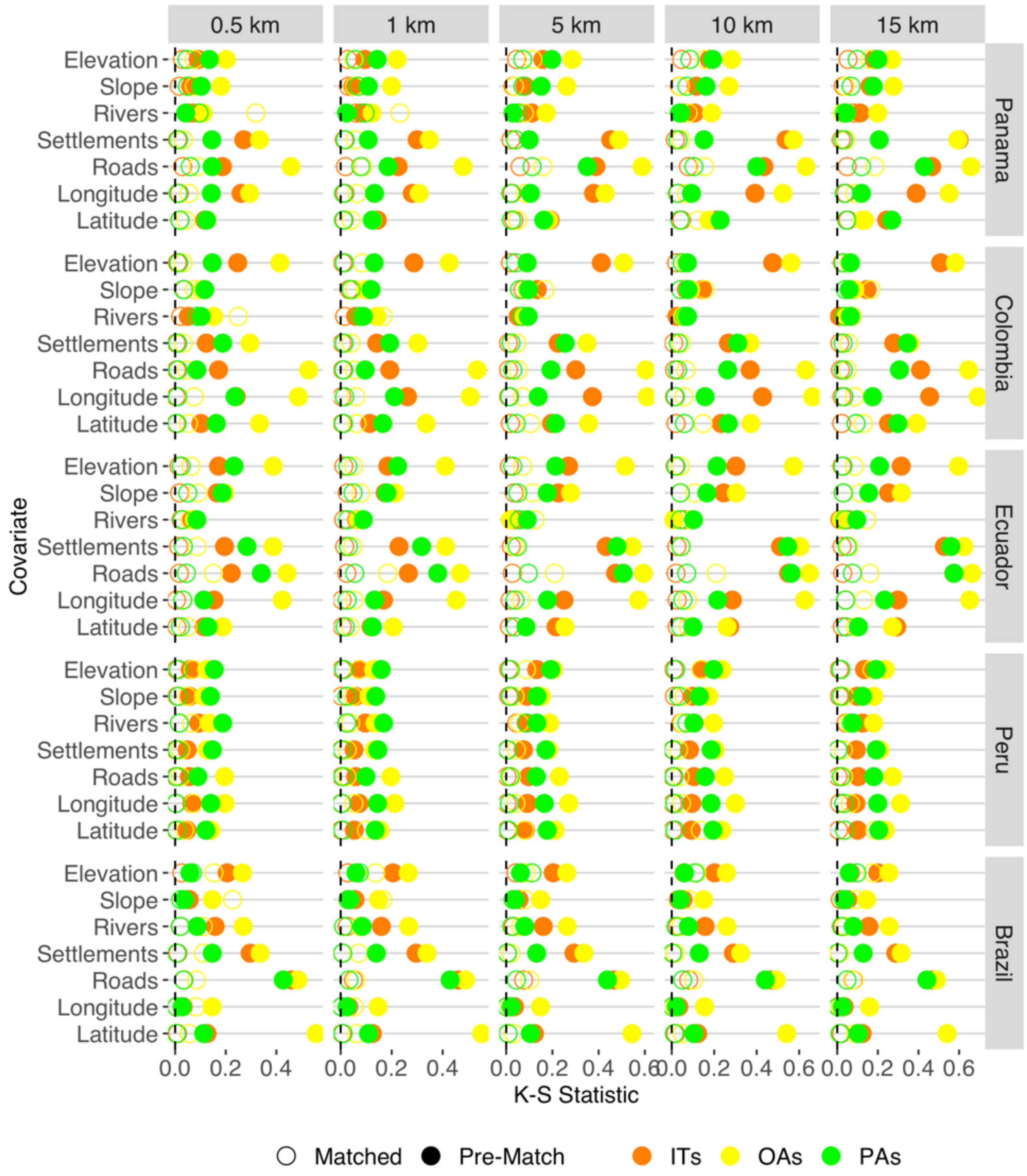

Fig E. Covariate falsification tests derived from linear mixed models in geographic discontinuity designs across the boundaries of ITs, OAs and PAs in neotropical countries.

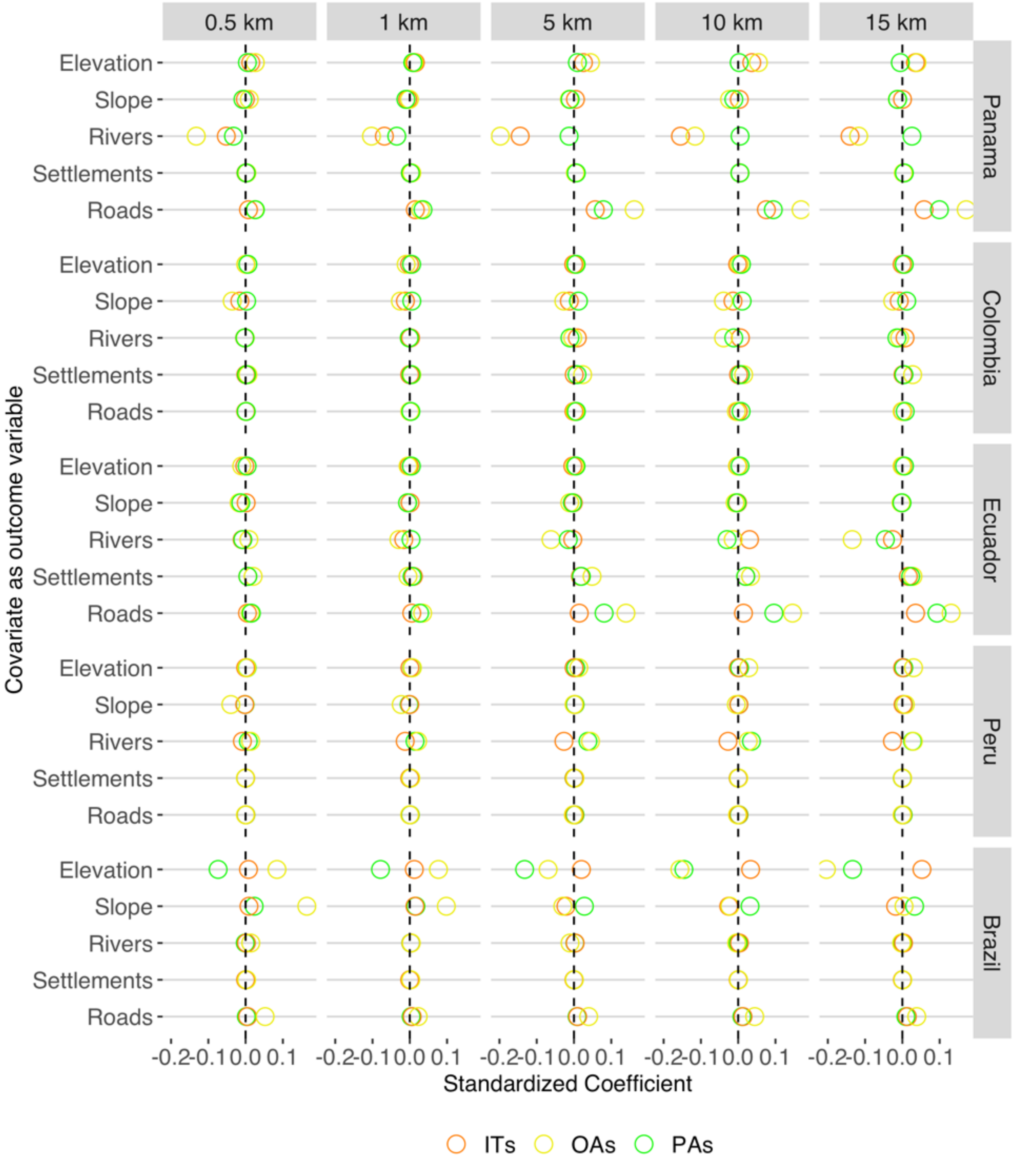

Supplement: S2 Appendix — (PDF) [file pone.0245110.s002.pdf]
