# Supplementary material for: Are indigenous territories effective natural climate solutions? A neotropical analysis using matching methods and geographic discontinuity designs
Source: PLoS One. 2021 Jul 12;16(7):e0245110. doi: 10.1371/journal.pone.0245110 (PMC8274867; doi:10.1371/journal.pone.0245110)
Supplement: S3 Appendix — (PDF) [file pone.0245110.s003.pdf]

S3 Appendix. Sensitivity Analyses.

Fig A. Sensitivity analysis in the temporal effects of ITs, OAs, and PAs on carbon stocks in 2003 and 2016 across neotropical countries.

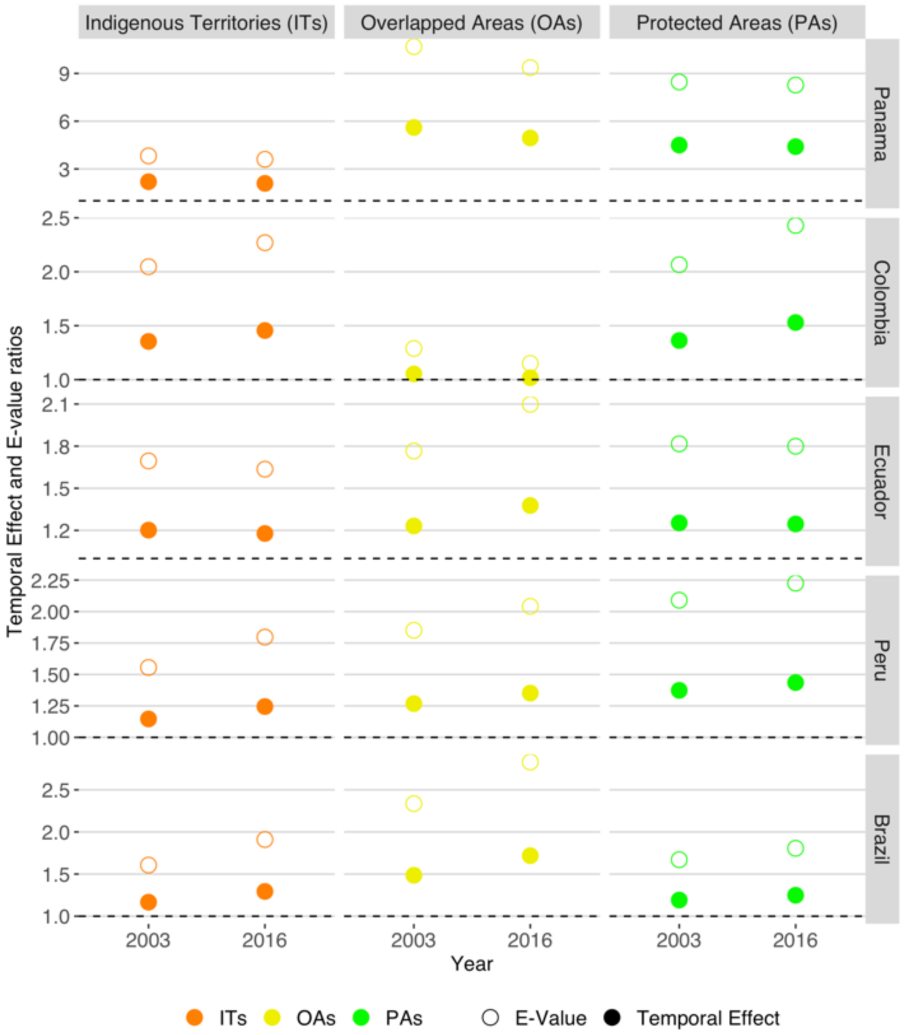

The temporal effect ratio is equivalent to the probability of a positive temporal effect in the treatment (i.e., ITs, OAs, and PAs) divided by the probability of a positive temporal effect in the control (other lands). The E-value represents the minimum strength that an unmeasured covariate would need to have with the treatment (i.e., ITs, OAs, PAs) and their temporal effect, for the treatments and temporal effect association not to be causal.

Fig B. Sensitivity analysis in the spatial effects of ITs', OAs', and PAs' boundaries on carbon stocks in 2003 and 2016 across neotropical countries.

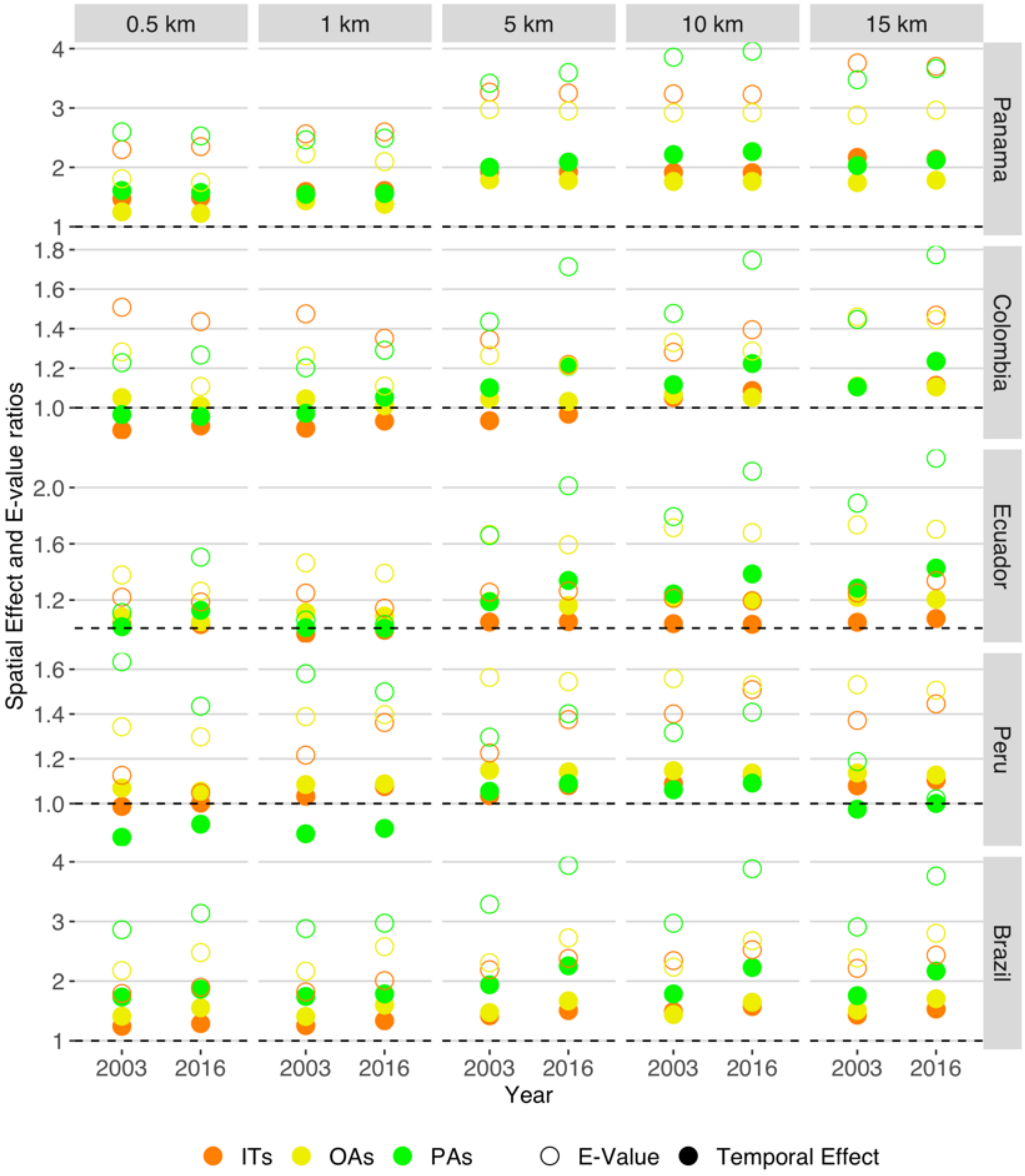

The spatial effect ratio is equivalent to the probability of a positive spatial effect in the treatment (i.e., ITs, OAs, and PAs) divided by the probability of a positive spatial effect in the control (other lands). The E-value represents the minimum strength that an unmeasured covariate would need to have with the treatment (i.e., ITs, OAs, PAs) and their spatial effect, for the treatments and spatial effect association not to be causal.
