# Supplementary material for: Are indigenous territories effective natural climate solutions? A neotropical analysis using matching methods and geographic discontinuity designs
Source: PLoS One. 2021 Jul 12;16(7):e0245110. doi: 10.1371/journal.pone.0245110 (PMC8274867; doi:10.1371/journal.pone.0245110)
Supplement: S4 Appendix — (PDF) [file pone.0245110.s004.pdf]

S4 Appendix. Temporal effects, carbon baselines, and human settlements distribution.

Fig A. The temporal effect of ITs, OAs, and PAs on carbon stocks between 2003 and 2016 across neotropical countries.

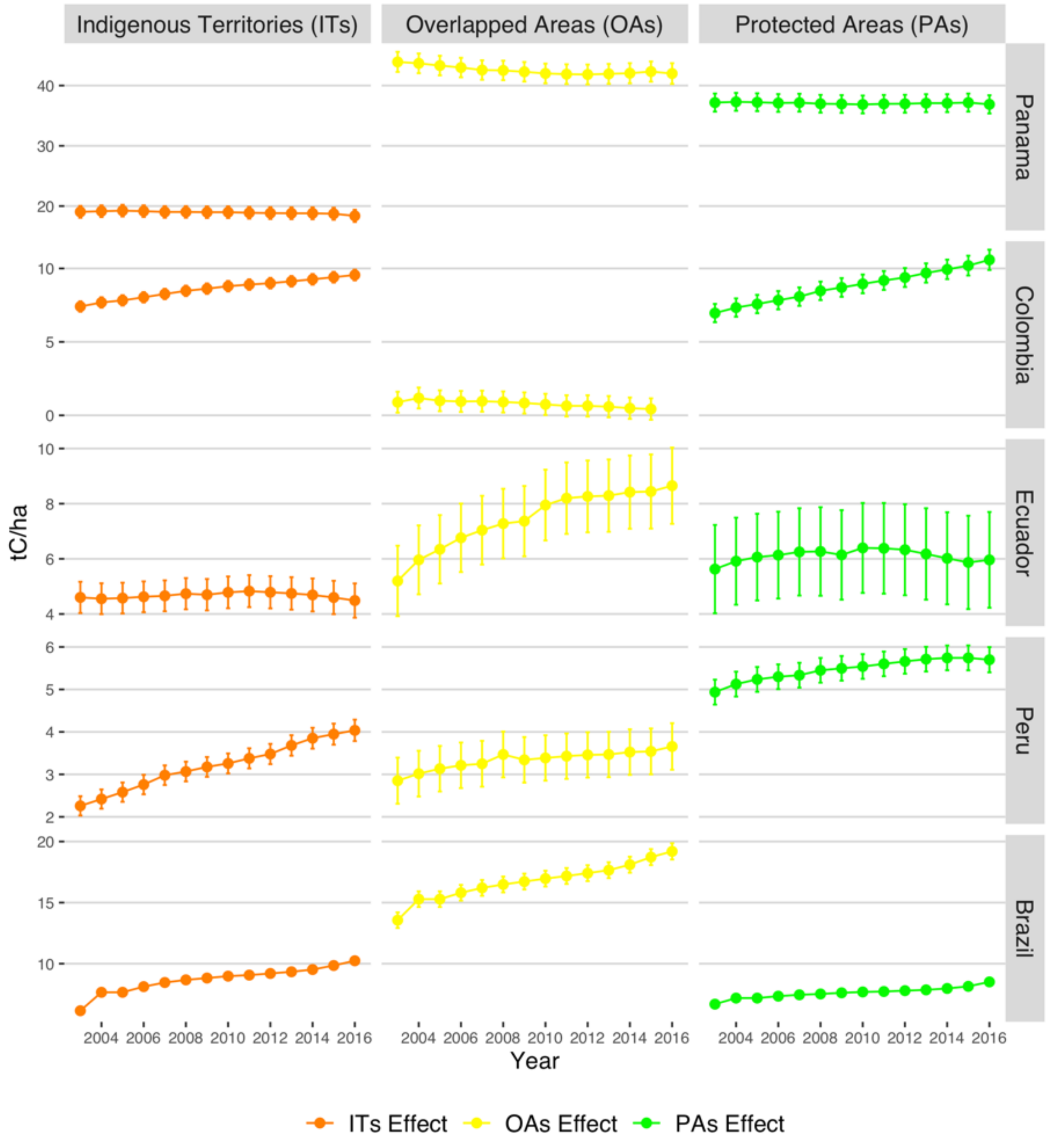

Each point are the significant annual effects ($p < 0.05$) of ITs (orange), OAs (yellow), and PAs (green) on carbon stocks. The temporal effects represent the annual differences of carbon stocks between ITs, OAs, and PAs with other lands after controlling for the spatial location through matching analysis and linear mixed

models. Error bars reflect 95% confidence intervals for the temporal effect derived from the linear mixed models.

Fig B. **The carbon stocks baseline of ITs', OAs', and PAs' temporal effects across neotropical countries.**

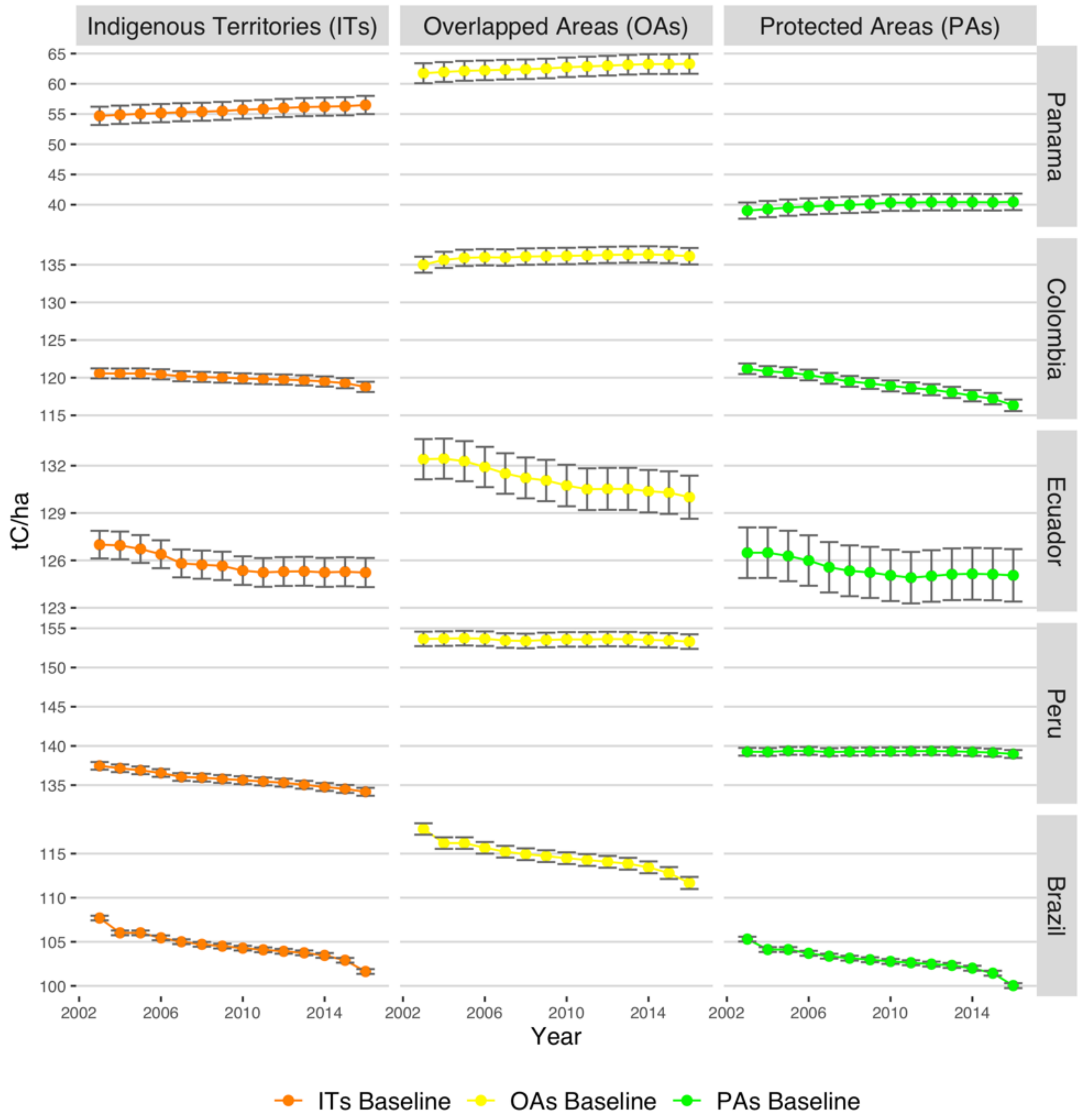

Each point represents the mean annual carbon stocks found in other lands (i.e., carbon stocks baseline) that share spatial location covariates with ITs (orange), OAs (yellow), and PAs (green) after matching analysis and

linear mixed models. Error bars reflect 95% confidence intervals for the carbon stocks baselines derived from the linear mixed models.

Fig C. Distribution of human settlements inside the boundaries of ITs, OAs, and PAs across neotropical countries.

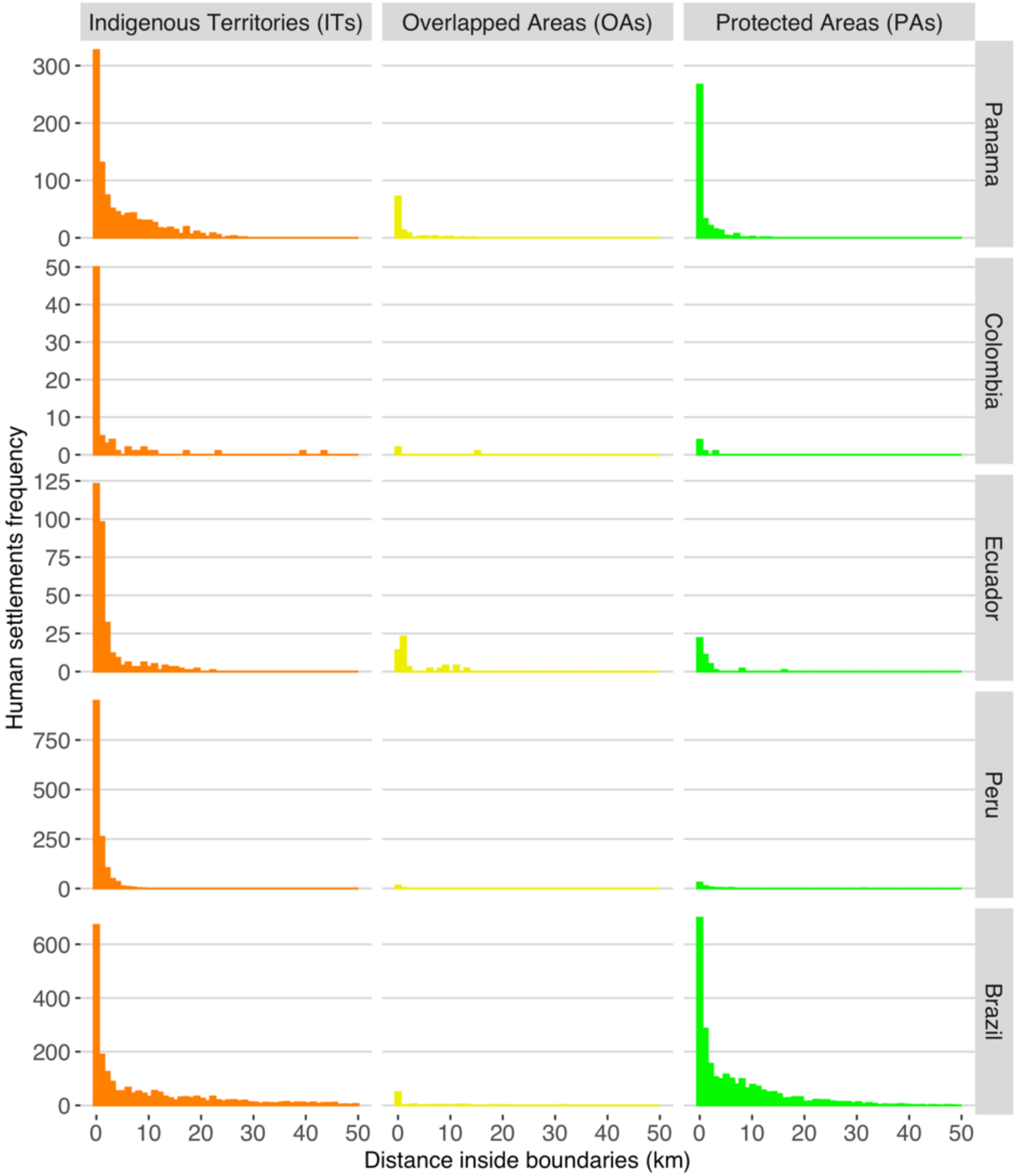

The human settlements included are those registered by national institutions in Amazon Basin countries and STRI in Panama. Data sources are shown in the S1 Appendix.

Fig D. The carbon stocks baseline outside the boundaries of ITs, OAs, and PAs across neotropical countries.

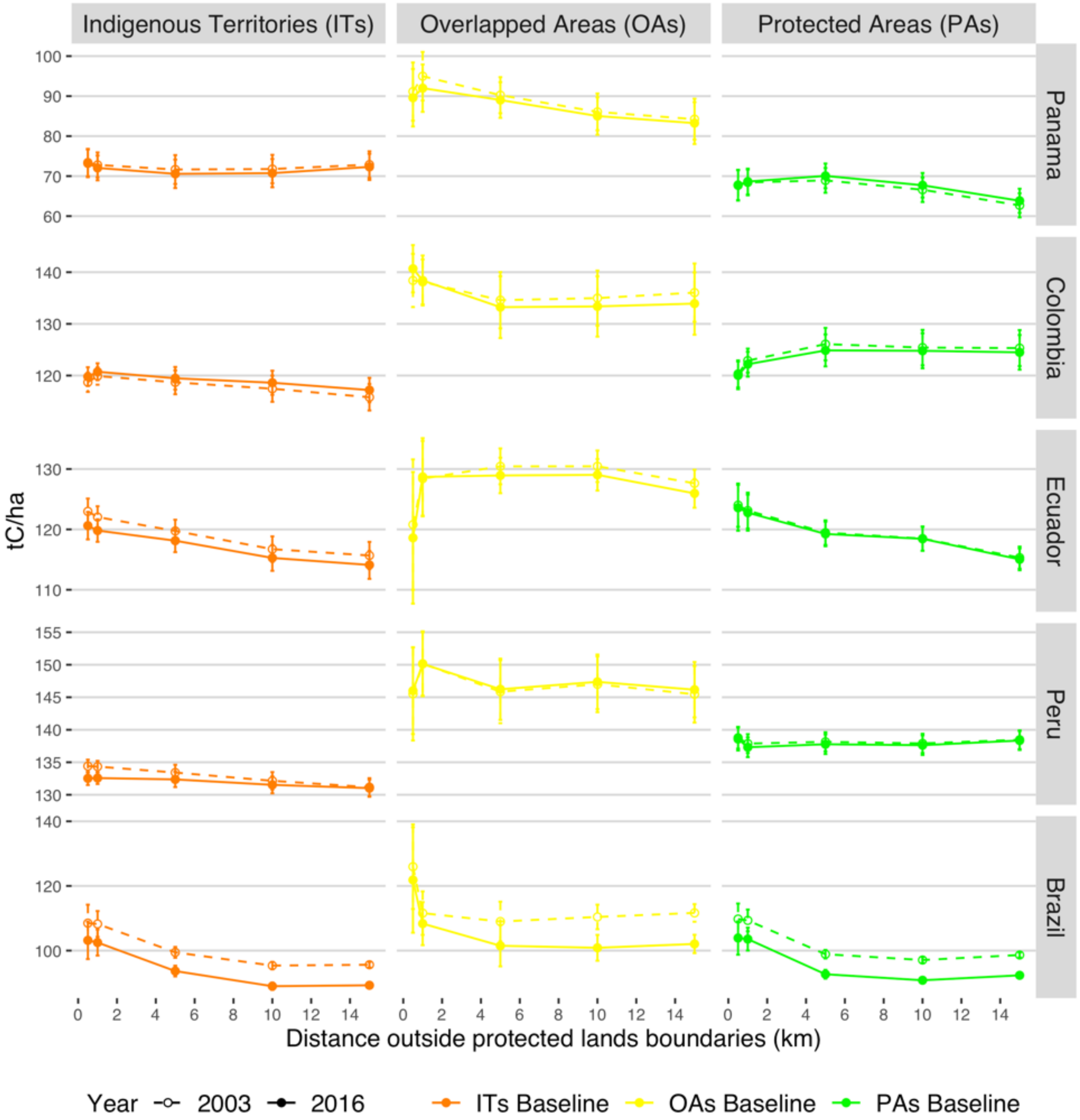

Full (2016) or empty (2003) points represent the mean annual carbon stocks found in other lands (i.e., carbon stocks baseline) outside the boundaries of ITs (orange), OAs (yellow), and PAs (green) at a certain buffer distance. Error bars reflect 95% confidence intervals for the carbon stocks baselines derived from linear mixed models.
